# A systematic methodological evaluation of sepsis guidelines: Protocol for quality assessment and consistency of recommendations

**DOI:** 10.1111/aas.70036

**Published:** 2025-05-13

**Authors:** Marwa Amer, Morten Hylander Møller, Anders Granholm, Haifa F. Alotaibi, Shadan AlMuhaidib, Zainab Al Duhailib, Amr Arafat, Michelle S. Chew, Marius Rehn, Martin Ingi Sigurðsson, Maija‐Liisa Kalliomäki, Klaus T. Olkkola, Ville Jalkanen, Wojciech Szczeklik, Hassan M. Alshaqaq, Kimberley Lewis, Kallirroi Laiya Carayannopoulos, Kimia Honarmand, Dipayan Chaudhuri, Mustafa Alquraini, Yasser S. Amer, Fayez Alshamsi, Waleed Alhazzani

**Affiliations:** ^1^ Medical/Critical Pharmacy Division King Faisal Specialist Hospital and Research Center Riyadh Saudi Arabia; ^2^ College of Medicine Alfaisal University Riyadh Saudi Arabia; ^3^ Department of Intensive Care Copenhagen University Hospital—Rigshospitalet Copenhagen Denmark; ^4^ Department of Clinical Medicine University of Copenhagen Copenhagen Denmark; ^5^ Section of Biostatistics, Department of Public Health University of Copenhagen Copenhagen Denmark; ^6^ Health Research Center Directorate General of Armed Forces Medical Services Riyadh Saudi Arabia; ^7^ Independent Researcher Riyadh Saudi Arabia; ^8^ Critical Care Medicine Department King Faisal Specialist Hospital and Research Centre Riyadh Saudi Arabia; ^9^ Department of Perioperative Medicine and Intensive Care Karolinska University Hospital Stockholm Sweden; ^10^ Division of Prehospital Services, Air Ambulance Department Oslo University Hospital Oslo Norway; ^11^ The Norwegian Air Ambulance Foundation Oslo Norway; ^12^ Institute of Clinical Medicine University of Oslo Oslo Norway; ^13^ Faculty of Medicine University of Iceland Iceland; ^14^ Division of Anaesthesia and Intensive Care Medicine Landspitali—The National University Hospital of Iceland Iceland; ^15^ Department of Anaesthesia Tampere University Hospital Tampere Finland; ^16^ Department of Anaesthesiology, Intensive Care and Pain Medicine University of Helsinki and Helsinki University Hospital Helsinki Finland; ^17^ Department of Intensive Care Medicine Tampere University Hospital Tampere Pirkanmaa Finland; ^18^ Centre for Intensive Care and Perioperative Medicine Jagiellonian University Medical College Kraków Poland; ^19^ Anesthesia and Intensive Care Department 5th Military Hospital Kraków Poland; ^20^ Emergency Medicine Department King Saud University Medical City Riyadh Saudi Arabia; ^21^ Department of Medicine McMaster University Hamilton Ontario Canada; ^22^ Department of Health Research, Methods, Evidence, and Impact McMaster University Hamilton Ontario Canada; ^23^ Research Institute of St Joseph's Healthcare Hamilton Hamilton Ontario Canada; ^24^ Division of Critical Care, Department of Medicine Mackenzie Health Vaughan Ontario Canada; ^25^ Emergency Medicine/Critical Care/NeuroCritical Care Department Almana Hospitals Dammam Saudi Arabia; ^26^ Pediatrics Department King Khalid University Hospital Riyadh Saudi Arabia; ^27^ Clinical Practice Guidelines and Quality Research Unit, Quality Management Department King Saud University Medical City Riyadh Saudi Arabia; ^28^ Research Chair for Evidence‐Based Health Care and Knowledge Translation King Saud University Riyadh Saudi Arabia; ^29^ Alexandria Center for Evidence‐Based Clinical Practice Guidelines Alexandria University Alexandria Egypt; ^30^ Adaptation Working Group Guidelines International Network Perth Scotland UK; ^31^ Department of Internal Medicine, College of Medicine and Health Sciences United Arab Emirates University Alain United Arab Emirates; ^32^ Department of Critical Care, College of Medicine King Saud University Riyadh Saudi Arabia; ^33^ Critical Care and Internal Medicine Department, College of Medicine Imam Abdulrahman Bin Faisal University Saudi Arabia

**Keywords:** AGREE II, clinical practice guidelines, consistency, guideline quality assessment, sepsis, systematic review

## Abstract

**Background:**

Sepsis is a leading cause of mortality worldwide, characterized by a dysregulated host response to infection. Despite the development of multiple clinical practice guidelines (CPGs) to standardize sepsis management, substantial variability exists in methodological quality and key clinical recommendations. This inconsistency complicates guideline implementation and potentially affects patient outcomes. The proposed systematic methodological review aims to evaluate the quality and consistency of sepsis guidelines to identify areas for improvement and provide actionable insights for guideline developers.

**Methods:**

This protocol outlines a systematic methodological review of sepsis CPGs published over the last two decades (2004–2025). A comprehensive search strategy will be conducted across PubMed, EMBASE, the Cochrane Library, and the official websites of professional societies to identify relevant guidelines. The inclusion criteria are CPGs targeting adult sepsis management published by recognized medical or governmental organizations with detailed methodological descriptions. We will use the Appraisal of Guidelines for Research and Evaluation II instrument to assess methodological quality across six domains: scope and purpose, stakeholder involvement, rigor of development, clarity of presentation, applicability, and editorial independence. Data extraction will focus on key clinical recommendations, including fluid resuscitation, antimicrobial therapy, vasopressor and inotrope use, corticosteroids, source control, blood glucose management, hemodynamic management, and mechanical ventilation management. The consistency of the recommendations will be analyzed, and trends in guideline quality over time will be evaluated. Artificial intelligence (AI) tools will be evaluated for data extraction processes in systematic reviews to determine their capacity for efficiency and accuracy in extracting data compared to human‐driven methods.

**Conclusion:**

By systematically appraising the quality and consistency of sepsis guidelines, this review aims to address the existing gaps and discrepancies in guideline development and application. These findings will provide valuable insights into the evolution of sepsis guideline quality, highlight areas for improvement, and support the development of more robust evidence‐based recommendations. These results will inform clinicians and guideline developers, ultimately enhancing the standardization and effectiveness of sepsis management worldwide. Integrating AI into the review process represents a novel methodological advancement that streamlines data extraction and analysis.

## 1 INTRODUCTION

Sepsis, a life‐threatening condition caused by dysregulated host response to infection, remains the leading cause of mortality among patients who are critically ill worldwide. Its complex pathophysiology and clinical heterogeneity make early recognition and effective management critical for improving patient outcomes.^1^ Clinical practice guidelines (CPGs) have long served as essential tools for standardizing sepsis management; however, the existence of multiple guidelines with significant variability in both quality and recommendations often complicates their application across diverse healthcare settings.^2^, ^3^, ^4^

This variability is particularly pronounced in core clinical areas, such as fluid resuscitation, antimicrobial therapy, vasopressor and inotrope use, corticosteroids, source control, blood glucose management, hemodynamic management, and mechanical ventilation management. Differences in the guideline development processes, evidence appraisal methodologies, and regional priorities have led to discrepancies in recommendations, posing challenges for clinicians seeking evidence‐based care.^5^ Furthermore, the methodological shortcomings of some guidelines, such as inadequate stakeholder involvement and limited rigor of development, may undermine their applicability and acceptance in clinical practice.

To address these challenges, we propose a systematic methodological review to evaluate the methodological quality and consistency of recommendations in existing sepsis guidelines. Additionally, by comparing the consistency in key clinical recommendations across guidelines, we aim to identify areas of alignment and discrepancy, offering valuable insights for clinicians and guideline developers.

The study will furnish actionable insights to refine sepsis guidelines, highlighting the necessity for robust, evidence‐based recommendations that are clinically applicable. It will assess guideline quality, methodological rigor, and improvement areas over time, aiming to better align sepsis management with clinical demands and improve patient outcomes. Additionally, this study introduces the use of artificial intelligence (AI) tools in data extraction to compare the efficiency and accuracy of AI with traditional human‐driven methods in systematic reviews.

## 2 METHODS

This study will be reported in accordance with the Preferred Reporting Items for Systematic Reviews and Meta‐Analyses (PRISMA) guidelines.^6^, ^7^ The protocol was registered in PROSPERO on January 26, 2025 (protocol ID: CRD42025639022).

### 2.1 Eligibility criteria

We will include guidelines that address sepsis or septic shock management in adults aged 18 years or older. Guidelines must provide key recommendations related to one or more core aspects of sepsis care, including fluid resuscitation, antimicrobial therapy, vasopressor use, corticosteroids, source control, blood glucose management, hemodynamic management, and mechanical ventilation management. These domains reflect the fundamental components of sepsis treatment strategies and serve as the primary focus for data extraction. Eligible guidelines must be published in English by recognized medical or governmental organizations, such as the World Health Organization (WHO), Society of Critical Care Medicine (SCCM), the National Institute for Health and Care Excellence, Centers for Disease Control and Prevention, or European Society of Intensive Care Medicine (ESICM). They may include consensus statements, position papers, or expert opinions with explicitly stated recommendations, provided they meet minimum development standards. Guidelines published between 2004 and 2025 are eligible for inclusion. This includes the Surviving Sepsis Campaign guidelines relevant to the management of critically ill adults with coronavirus disease 2019, given their importance in sepsis management.^8^ Guidelines addressing specific aspects of sepsis care, such as fluid therapy (e.g., ESICM guidelines), will also be considered. Additionally, guidelines focused on the management of sepsis and septic shock in special populations, such as maternal sepsis, cancer patients, and febrile neutropenia, will be included. Precision medicine guidelines in sepsis will be included if they encompass key recommendations for core aspects of sepsis care. Similarly, guidelines on procalcitonin use in sepsis will be considered if they provide recommendations directly applicable to sepsis management, particularly for antimicrobial therapy decisions.

Guidelines with substantive revisions, contextual adaptations, or updated recommendations to align with specific regional practices or resource settings will also be eligible, provided they meet the defined eligibility criteria and include detailed methodologies.

Guidelines will be excluded if they:

1. Lack of accessible methodologies or explicit recommendations.
2. Are available solely as executive summaries.
3. Consist only of errata—published corrections or clarifications to previously released guidelines. While errata may address minor errors or omissions, they do not represent standalone guidelines and often lack the necessary context for independent assessment. However, errata clarifying or updating information within eligible guidelines will be integrated into the review of the original guideline to ensure accuracy and completeness during data extraction.
4. Are available solely as endorsement documents by a society but originally developed and published by another organization without substantial modifications.
5. Guidelines that primarily address broader topics related to critically ill adults, without explicit emphasis on sepsis or septic shock, will be excluded unless they contain specific sections or recommendations directly applicable to sepsis management.
6. Guidelines focusing on alternative or adjunctive therapies, such as herbal, traditional, or complementary medicine approaches. This does not apply to conventional adjunctive therapies like corticosteroids, which are part of standard sepsis management.
7. Guidelines focusing solely on sepsis‐induced disseminated intravascular coagulation or specific infections (e.g., meningitis and arthritis) without broader sepsis management strategies.

### 2.2 Data sources and searches

To identify relevant guidelines, a comprehensive search strategy will be implemented across major medical databases, including PubMed, EMBASE, and the Cochrane Library. Specialized platforms, such as Guideline Central and WHO repositories, will also be searched, alongside manual searches of individual organizational websites. The search strategy, developed in collaboration with experienced medical librarians, employs Boolean operators and Medical Subject Heading terms to ensure sensitivity and specificity. Keywords such as “sepsis,” “septic shock,” “clinical practice guidelines,” “fluid therapy,” “antibiotics,” and “vasopressors” will be used to capture both broad and specific aspects of sepsis guidelines. To maximize inclusivity, the search integrates two main concepts—Sepsis/Septic and a Guidelines filter—while incorporating additional terms such as “recommendations,” “statement,” “position papers,” “expert opinions,” “protocol,” “pathway,” and “consensus” to refine results without excluding relevant guidelines. Given the potential for broader interpretations of terms like “protocol” and “pathway,” these will be incorporated in proximity (within two terms) to “care,” “critical,” “clinical,” or “practice” to maintain relevance. Preliminary testing of the search strategies confirmed a balance between inclusivity and precision, minimizing irrelevant results while preserving comprehensiveness.

Search results will be exported to systematic review management software for screening and data extraction. Iterative refinements of search terms will be applied as needed to validate the strategy and ensure the robust retrieval of relevant guidelines (Appendix S1).

### 2.3 Study selection

**FIGURE 1:**
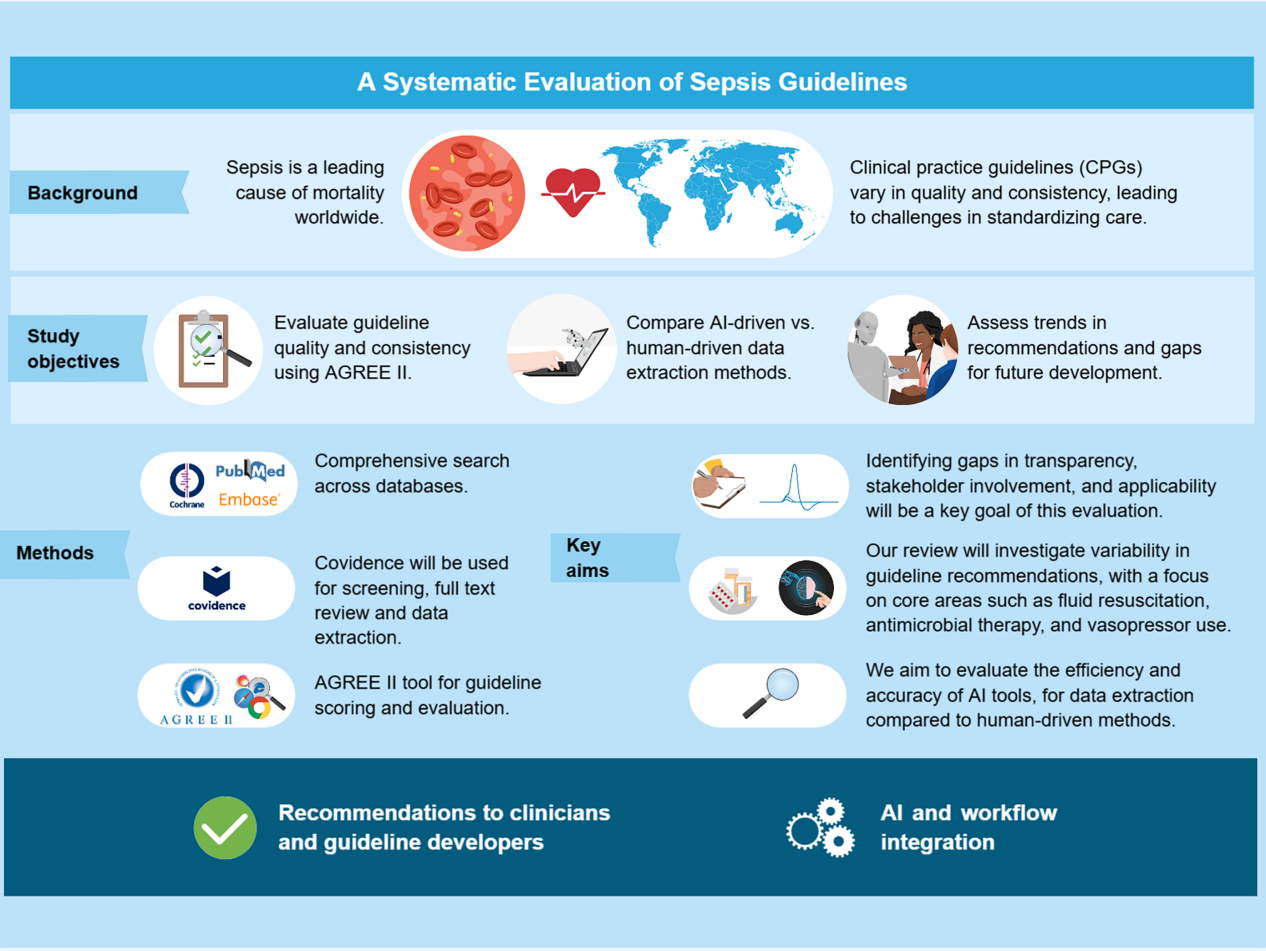
Visual abstract. The inclusion of multiple guideline versions allows the assessment of trends in quality and consistency over time. Discrepancies in recommendations may reflect evidence‐driven updates rather than methodological differences.

All identified guidelines will undergo a rigorous screening process independently and in duplicate by two reviewers. This process includes a systematic evaluation of titles, abstracts, and full texts to ensure alignment with the inclusion criteria. Any discrepancies between reviewers will be resolved by consulting a third reviewer to maintain objectivity and consistency (Figure 1).

### 2.4 Data extraction

Two reviewers, independently and in duplicate, will extract key information from each included guideline. All the extracted data are cross‐verified to ensure consistency and accuracy. Table 1 presents the detailed data extraction process, including guideline features, populations, and key recommendations.

**TABLE 1:** Data extraction form.

Guideline features *Title*: full title of the guideline. *Authoring organization*: name of authoring body (e.g., SCCM, WHO, ESICM). *Year of publication*: the year the guideline was published. *Country/region*: origin or region of publication. *Version*: version of the guideline (e.g., new, updated). Population characteristics *Clinical scope*: scope of conditions covered (e.g., sepsis, septic shock). *Disease focus*: focus areas such as COVID‐19 management. Methodology and development *Development method*: methodology used (e.g., evidence‐based, consensus). *Grading system to assess the certainty of evidence or approaches used in evidence synthesis and decision‐making* (e.g., GRADE, USPSTF, RAND/UCLA Appropriateness Methodology, Delphi Method) Key recommendations and consistency analysis Fluid resuscitation ○ Type (e.g., colloid, crystalloid), timing, and volume. Antimicrobial therapy ○ Antibiotic type, timing, and duration. Vasopressors and inotropes ○ Type, timing. Corticosteroids ○ Indications, dosage, and duration. Source control ○ Strategies and timing for controlling infection sources (e.g., surgery, drainage) Blood glucose management ○ Glycemic targets and strategies for glucose control. Hemodynamic management Hemodynamic targets (e.g., MAP, cardiac output, systemic vascular resistance), use of advanced hemodynamic monitoring techniques (e.g., pulse contour analysis, thermodilution methods), fluid responsiveness assessment (passive leg raise, stroke volume variation), lactate Mechanical ventilation management Ventilation strategies (e.g., lung‐protective ventilation, tidal volume, PEEP, recruitment maneuvers), modes of ventilation, oxygenation targets (PaO_2_/FiO_2_ ratio, SpO_2_ goals), and the role of adjunctive strategies (e.g., prone positioning, neuromuscular blockade, ECMO in refractory hypoxemia). Implementation and applicability *Resources required*: list of required resources or high‐resource settings. *Implementation limitations*: limitations in clinical practice (e.g., resource availability). *Monitoring and evaluation*: criteria for tracking outcomes and guideline adherence. Additional notes and observations *Funding sources*: funding sources and disclosures. *Conflicts of interest*: potential conflicts of interest disclosed. *Update frequency*: frequency of updates or revisions.

Abbreviations: COVID‐19, coronavirus disease 2019; ECMO, Extracorporeal Membrane Oxygenation; ESICM, European Society of Intensive Care Medicine; GRADE, Grading of Recommendations, Assessment, Development, and Evaluation; MAP, mean arterial pressure; PaO_2_/FiO_2_ ratio, partial pressure of arterial oxygen to fraction of inspired oxygen ratio; PEEP, positive end‐expiratory pressure; SCCM, Society of Critical Care Medicine; SpO_2_ goals, oxygen saturation goals; USPSTF, U.S. Preventive Services Task Force; WHO, World Health Organization.

### 2.5 Risk of bias assessments

We will assess the methodological quality of each included guideline using the Appraisal of Guidelines for Research and Evaluation II (AGREE II) tool, an internationally recognized framework designed to evaluate six key domains (Table 2). Four reviewers, including clinicians and methodologists with expertise in guideline development and evidence appraisal, will independently score each guideline using the My AGREE PLUS platform,^9^ which is a web‐based tool provided by the AGREE Trust. This platform facilitates structured, transparent, and collaborative appraisals, ensuring the rigor and efficiency of the assessment process.^10^

**TABLE 2:** AGREE II scoring.

Evaluation of six domains: *Scope and purpose*: objectives, health questions, and target population. *Stakeholder involvement*: representation of users and stakeholders. *Rigor of development*: methods for evidence gathering and updates. *Clarity of presentation*: format and ease of locating key recommendations. *Applicability*: implementation aspects, resource needs, and monitoring criteria. *Editorial independence*: evaluation of biases and conflicts of interest.

*Note*: 1. Scope and purpose (Items 1–3): evaluate objectives, specific healthcare issues, and intended population. 2. Stakeholder involvement (items 4–6): assesses the inclusivity and representation of developers. 3. Rigor of development (Items 7–14): examine evidence collection, synthesis, and methods for formulating recommendations. 4. Clarity of presentation (Items 15–17): evaluate the structure, format, and ease of locating recommendations. 5. Applicability (Items 18–21): considers implementation challenges, required resources, and monitoring criteria. 6. Editorial independence (Items 22–23): assesses independence from funding sources and conflicts of interest management.

Prior to the formal appraisals, all reviewers will undergo training to ensure familiarity with the AGREE II instrument and its platform functionality. The My AGREE PLUS platform enhances the review process by enabling collaborative scoring, where multiple reviewers can independently assess guidelines online while centrally storing scores and comments to minimize data loss or errors. It also improves transparency by documenting scores and justifications and offering a traceable audit trail for methodological rigor (Appendix S2). We will conduct a pilot assessment of the selected guidelines to ensure calibration across the reviewers before the full evaluation commences.

### 2.6 Data synthesis

The My AGREE PLUS platform will be used to streamline data analysis, support discrepancy resolution by highlighting variations in scores, and facilitate discussions among the reviewers to build a consensus. It automatically calculates domain means and summary scores, thus saving time and ensuring accuracy. The platform also generates customizable reports, including domain scores, inter‐rater reliability metrics, and reviewer comments, which can be directly incorporated into the final analysis and manuscript. Its centralized data storage and global accessibility reduce logistical challenges and enable international collaboration without the need for in‐person meetings.

Each of the six AGREE II domains will be assessed individually using a 7‐point Likert scale (1 = strongly disagree, 7 = strongly agree). Each guideline will be assessed independently by a group of four AGREE II assessors, with different sets of assessors evaluating different guidelines to ensure a balanced and diverse appraisal process. Any discrepancies of three points or more will be discussed to reach a consensus.

A score of one indicates substantial discordance or lack of information, whereas a score of seven represents full compliance with the AGREE II criteria. Intermediate scores (2–6) reflect partial satisfaction with the criteria.

Aggregate scores will be calculated across domains, and the guidelines are classified into three quality categories based on their scores.^11^, ^12^

- High quality: overall scores above 60% in at least five domains—recommended for use.
- Moderate quality: scores between 30% and 60% in most domains, which are recommended after revision to address identified gaps.
- Low quality: scores below 30% across domains, which is not recommended owing to methodological weaknesses.

Following the evaluation of all 23 AGREE II items, this systematic approach ensured transparency, consistency, and reliability in the quality assessment of sepsis guidelines, guiding their categorization and subsequent recommendations. We will also calculate the mean, median, and interquartile ranges (IQRs) for each domain across different guidelines (Figure S1, Tables S1 and S2).

#### 2.6.1 Subgroup analysis

To investigate variations in guideline quality, we will conduct subgroup analyses, comparing AGREE II scores across different categories.

1. International versus national guidelines: assess whether international guidelines exhibit higher quality scores than national guidelines. We hypothesize that international guidelines will exhibit higher quality scores compared to national guidelines based on the assumption that international guidelines often have access to broader resources, diverse expertise, and more extensive peer review processes, which may contribute to higher methodological rigor and clarity.
2. The Income Classification of Countries: guidelines are analyzed based on the World Bank income classification (latest 2023 data), grouping countries into high‐income, upper‐middle‐income, and lower‐middle‐income categories to explore the association between resource availability and guideline quality. The country income level will be determined a priori based on the country where the issuing society is primarily located. If no society is behind the guideline, the country of the corresponding author will be used for classification. We hypothesize that guidelines from high‐income countries will demonstrate higher quality scores. This hypothesis reflects the potential influence of greater resource availability, more robust healthcare infrastructure, and higher investment in research and development in high‐income countries.
3. Guidelines using the GRADE methodology versus those using other frameworks. We hypothesize that guidelines utilizing the GRADE methodology will demonstrate higher overall quality scores compared to those developed using alternative frameworks. This hypothesis is based on the assumption that GRADE provides a structured and transparent approach to evaluating evidence certainty and recommendation strength, leading to greater methodological rigor, consistency, and clarity in guideline development.

#### 2.6.2 Statistical approaches (quantitative synthesis)

We will evaluate differences in AGREE II scores across subgroups, such as international versus national guidelines and income classifications (high‐income, upper‐middle‐income, and lower‐middle‐income countries). To ensure a comprehensive analysis, we will consider both continuous data (e.g., raw AGREE II domain scores) and categorical classifications (e.g., high quality [>60%], moderate quality [30–60%], or low quality [<30%] based on overall AGREE II scores).

Trend tests will be conducted to identify potential patterns of quality improvement over time, focusing on whether subsequent versions of guidelines demonstrate systematic improvements in AGREE II domain scores (e.g., methodological rigor, stakeholder involvement, clarity of presentation) and overall quality. This will allow us to discern whether observed changes reflect progress in guideline development practices or variability due to other factors, such as differences in organizational resources or evidence synthesis approaches.

Additionally, we will explore whether particular domains consistently improve (e.g., methodology or applicability) or whether advancements are uneven across domains. By explicitly comparing trends in both continuous and categorical outcomes, we aim to identify areas where guideline development has progressed and highlight domains requiring further focus in future updates. Visualizations, such as line graphs for continuous data and heatmaps for categorical trends, will be used to depict these patterns and provide a clear representation of quality trajectories over time.

#### 2.6.3 Regression models

We will use separate and combined models to assess guideline quality.

1 *Separate models*: These models allow for domain‐specific insights into predictors of overall quality.
  ○ *Linear regression*: If the overall AGREE II score is treated as a continuous variable, we will use univariable linear regression to assess the association between each individual factor and overall guideline quality.
  ○ *Logistic regression*: If the overall AGREE II score is categorized as binary (high quality: >60%; moderate/low quality: ≤60%), we will use univariable logistic regression to evaluate associations between each factor and high‐quality guidelines.
2 *Combined models*: These models provide a holistic view of how predictors collectively influence guideline quality.
  ○ All independent variables (listed below) will be included in multivariable linear and logistic regressions to assess, regardless of their significance in univariable analyses in line with best statistical practice^13^
1 *Publication year*—To evaluate whether more recent guidelines exhibit higher quality.
2 *Funding status and sources*—Whether the guideline was funded and, if so, whether the funding source was an external organization, government body, professional society, or industry funding.
3 *AGREE II domains*—To assess the impact of specific methodological components on overall guideline quality.
4 *Presence and number of methodologists*—Whether methodologists were involved in guideline development and, where available, the number of methodologists contributing.
5 *Country income level*—Categorized based on the World Bank classification (high‐income, upper‐middle‐income, and lower‐middle‐income countries).
6 *Geographic region*—Defined based on predefined categories (e.g., North America, MENA, Europe), determined a priori by the organization responsible for developing the guideline. If the organization is multinational or unclear, classification will be determined using the first author's affiliation and, if necessary, the funding body.
7 *Stakeholder involvement*—Whether the guideline included engagement with key stakeholders such as patients, practitioners, or policymakers.
8 *Conflict of interest disclosure*—Whether a conflict‐of‐interest statement was reported (yes/no).
  ○ *LASSO regression*: To address potential multicollinearity among AGREE II domains, we will use LASSO regression, which penalizes less informative variables and retains only the most relevant predictors. By using LASSO regression, we account for interdependencies among AGREE II domains, mitigating statistical challenges related to multicollinearity.

The choice of regression model—linear regression for continuous outcomes, univariable and multivariable logistic regression for binary outcomes, or LASSO regression to address multicollinearity—ensures analytical rigor by adapting to the specific nature of the dependent variable.

We will use the intraclass correlation coefficient (ICC) to assess scoring consistency among the four AGREE II reviewers. This approach provides a robust measure of agreement when multiple raters evaluate continuous data.

Key reliability metrics:

- ICC threshold: a value of ≥0.80 indicates strong reliability and agreement among reviewers for continuous data (e.g., domain scores).
- Kendall's *W* coefficient: will be used as a secondary measure to evaluate ranking consistency when ordinal data analysis is required.

This combination of methods ensured rigor and robustness in evaluating inter‐rater reliability while addressing scoring variability across domains.

#### 2.6.4 Narrative synthesis

The qualitative narrative synthesis complements quantitative analyses by identifying patterns in guideline quality and recommendation consistency. This approach provides contextual insights into variations in guideline development, methodological rigor, and clinical recommendations.

Key focus areas include:

1. *AGREE II scores*: The synthesis will describe variations in domain scores (e.g., consistently high scores in clarity of presentation versus lower scores in stakeholder involvement) and explore factors contributing to these differences. Particular attention will be given to the domains of methodology, conflicts of interest, and applicability.
2. *Recommendation consistency*: Patterns of alignment and divergence across guidelines will be examined, highlighting areas of strong consensus (e.g., antibiotic timing) and notable variability (e.g., corticosteroid use and vasopressor initiation). Recommendations will be classified as supporting, opposing, or neutral based on their alignment with clinical practices. Strengths will be assessed based on the level of supporting evidence (e.g., high‐quality randomized controlled trials vs. expert consensus).^14^ Any observed discrepancies in recommendations will be evaluated in the context of advancements in underlying evidence and shifts in clinical practice. This ensures that differences are not solely attributed to methodological rigor but also take into account evolving scientific knowledge.

### 2.7 Use of AI

An independent coauthor who is not involved in manual data extraction will use advanced language models to evaluate the capacity of AI tools for data extraction, efficiency, and accuracy compared to human‐driven methods.^15^ Our approach mirrors that of a recent study in *Acta Anesthesiologica Scandinavica* on personalized perioperative pain management.^16^ The final eligible articles for data extraction will be uploaded to the latest version of ChatGPT (GPT‐4, Open AI, San Francisco, USA). ChatGPT will be instructed to extract variables, such as author, publication year, target population, and key recommendations, within each guideline, conduct AGREE II domain assessments, and provide comments within each domain. AI‐extracted data and extraction time will be documented and compared to human/manual extraction time. A single coauthor has standardized the prompts given to the AI to ensure consistency (Table S3).

### 2.8 Data synthesis of AI output

AI‐driven data extraction will be quantitatively and qualitatively evaluated against human‐driven methods in the context of a sepsis guideline review, focusing on efficiency gains, accuracy, and error analysis, and consistency in extraction.

The time required for data extraction is tracked separately for both the AI‐ and human‐driven methods. Metrics such as the mean time per guideline and total time for data extraction will be compared. The percentage of time saved by the AI tools will be calculated to quantify the efficiency gains. For instance, if AI reduces the extraction time for a dataset by 50%, this improvement will be highlighted in bar charts or tables.

Discrepancies between the AI‐ and human‐extracted data will be systematically recorded and categorized into omissions, misclassifications, or extraction errors. Accuracy metrics, including precision and recall, will be calculated to assess AI's ability to reliably extract information. Error rate comparisons will identify the data types (e.g., AGREE II scores and core recommendations) that are most prone to AI errors.

The consistency between the AI and human approaches will be evaluated based on the type of data. We will assess the numerical variables (e.g., AGREE II scores) using Pearson's correlation coefficient to measure the linear correlation between the AI and human evaluations. We will analyze ordinal or ranked data (e.g., recommendation strength) using Spearman's rank correlation to evaluate whether AI preserved the relative ranking order established by human reviewers. The results will be presented as scatterplots with correlation coefficients and heat maps to visually depict the alignment between the AI‐ and human‐driven extractions, highlighting areas of strong agreement or divergence.

## 3 DISCUSSION

Sepsis guidelines emphasize timely interventions, including antimicrobial therapy and source control, which are essential for improving outcomes.^17^ Heterogeneity in sepsis presentation highlights the need for individualized management that accounts for factors such as comorbidities and physiological variability.^18^, ^19^, ^20^, ^21^, ^22^

The variability in methodological quality and consistency of current sepsis CPGs presents important challenges for clinicians and developers. Core recommendations on fluid resuscitation, antimicrobial therapy, and vasopressor use show inconsistencies, impacting standardized, evidence‐based care delivery. These variations may cause disparities in clinical decisions and patient outcomes across different healthcare settings. This systematic methodological evaluation systematically evaluates these guidelines to offer insights for enhancing their quality, transparency, and practical applicability.

This protocol provides clinicians with a key tool for identifying and prioritizing guidelines that follow rigorous, systematic methodologies. It highlights guidelines developed through transparent processes and supported by robust evidence to mitigate inconsistencies and enhance clinical decision‐making. To further facilitate the identification of high‐quality guidelines, we will include a summary table in the final manuscript that categorizes guidelines based on their adherence to methodological rigor, transparency, and supporting evidence. This will provide a structured overview to aid clinicians in selecting the most reliable guidelines for sepsis management. By ensuring the guidelines are relevant and practical, clinicians can provide more consistent and effective care, ultimately improving patient outcomes. The emphasis on transparency and systematic methodologies ensures these guidelines are evidence‐based, user‐friendly, and adaptable to real‐world scenarios.

The findings highlight the necessity for guideline developers to utilize structured methods to enhance methodological rigor, clarity, and relevance. Employing transparent processes for evidence retrieval and evaluation aids in creating trustworthy recommendations. Thorough evidence reviews and precise citations enhance traceability, allowing users to verify the basis of recommendations, thereby promoting trust and usability. Ensuring alignment with established quality assessment tools, such as AGREE II, helps evaluate the methodological soundness and transparency of guidelines. Involving a range of stakeholders, such as clinicians, patients, and other end‐users, increases the guidelines' relevance and acceptance. Addressing conflicts of interest and including expert reviews enhances credibility and integrity, supporting the creation of robust and impactful future guidelines.

This study includes all versions of guidelines published from 2004 to 2025 to evaluate trends in guideline quality over time. We aim to assess how the quality and methodological rigor of guidelines evolve with each iteration. By analyzing multiple versions, we gain insights into the effectiveness of updates and revisions in addressing gaps, incorporating new evidence, and aligning with standards like AGREE II. Our analysis will also explore discrepancies in recommendations in relation to advancements in scientific evidence and changes in clinical practice. This comprehensive evaluation will inform future updates to sepsis guidelines.

Recent advancements in AI‐driven evidence synthesis tools, such as ChatGPT and Elicit, have shown promising results in reducing the workload and increasing consistency during literature screening and qualitative data extraction. Table S4 summarizes the AI tools used for the systematic reviews.^23^ ChatGPT was chosen for its accessibility, affordability, and high accuracy, with 96% specificity and 93% sensitivity in screening tasks.^24^ Its versatility supports multiple phases of systematic reviews—research question formulation, data extraction, synthesis, and even academic writing—while enabling customization to adapt to various research methodologies. ChatGPT's integration potential with tools such as Covidence enhances performance, and its design encourages human–AI collaboration to minimize biases and errors. Given its scalability and adaptability, it is a cost‐effective solution for supporting data extraction in systematic reviews. Future research should continue to benchmark AI tools to refine workflows and improve their integration into evidence synthesis frameworks.

The integration of AI tools such as ChatGPT into systematic review workflows has limitations. AI tools depend on precise prompts, with variations affecting outputs, thus necessitating standardized prompts and iterative testing for consistent results. These tools might misinterpret complex recommendations, requiring human oversight for verification and ambiguity resolution. They may also lack context, struggle with nuanced interpretations, and face transparency issues due to proprietary algorithms. Additionally, biases in the training data necessitate manual validation to ensure reliability. Language barriers can impede accurate data extraction from non‐English guidelines. The variability in time‐tracking for AI‐ and human‐driven data extraction suggests the need for future studies to investigate standardized tracking methods, including built‐in timers in AI tools, to enhance performance and reproducibility.

By adopting standardized tracking and validation methods, future research can provide robust and reproducible metrics to validate the efficiency of AI and inform its broader implementation in systematic reviews.

This protocol lays the groundwork for a comprehensive and systematic methodological evaluation of adult sepsis guidelines, focusing on both methodological quality and consistency of key clinical recommendations. The structured application of the AGREE II tool and detailed consistency analyses will generate actionable insights for clinicians and guideline developers.

Employing a combined quantitative and qualitative approach, this study ensures a robust evaluation framework. The AGREE II instrument offers a standardized measure of guideline quality, and consistency analyses illuminate areas of alignment and discrepancy across essential clinical domains. Together, these findings will inform future updates to the sepsis guidelines, encouraging the development of transparent, methodologically sound, and clinically relevant recommendations.

One limitation of this review is the exclusion of non‐English guidelines, which may have led to the omission of relevant sepsis guidelines developed in other languages. This decision was made to ensure consistency in methodological assessment and feasibility in data extraction, as translation complexities could introduce bias or misinterpretation. While this approach allows for a standardized evaluation of guideline quality using the AGREE II instrument, it may limit the generalizability of findings to non‐English‐speaking regions. Future research should consider multilingual assessments or translation methods to capture a more comprehensive global perspective on sepsis guidelines.

Ultimately, this review supports clinicians in delivering consistent, evidence‐based care while equipping guideline developers with tools to create impactful and practical sepsis guidelines. These efforts will contribute to reliable and effective sepsis management strategies, thereby improving patient outcomes across diverse healthcare contexts.

## AUTHOR CONTRIBUTIONS

Dr. Marwa Amer and Prof. Waleed Alhazzani led this project. Both project leads were responsible for conceptualizing the study, developing the protocol, and overseeing the project. The screening, full‐text review, and data extraction processes are conducted using Covidence and managed by Haifa Al‐Otaibi, Zainab Al Duhailib, Hassan M. Alshaqaq, Shadan AlMuhaidib, and Marwa Amer. The AGREE II evaluations to assess guideline quality will involve contributions from Marwa Amer, Waleed Alhazzani, Ville Jalkanen, Wojciech Szczeklik, Kimberley Lewis, Kallirroi Laiya Carayannopoulos, Kimia Honarmand, Dipayan Chaudhuri, Mustafa Alquraini, Yasser S. Amer, Fayez Alshamsi, Anders Granholm, Martin Ingi Sigurðsson, Marius Rehn, Michelle S Chew, Maija‐Liisa Kalliomäki, Klaus T. Olkkola, Morten Hylander Møller, Zainab Al Duhailib, and Amr Arafat. Statistical analyses will be performed by Amr Arafat and Shadan AlMuhaidib. Amr Arafat reviewed the AI sub‐proposal methodological approach. All authors contributed to the development and review of the manuscript and approved the final version for submission.

## FUNDING INFORMATION

None.

## CONFLICT OF INTEREST STATEMENT

Waleed Alhazzani, Morten Hylander Møller, Fayez Alshamsi, Kimberley Lewis, and Dipayan Chaudhuri were members with conflicts of interest (involvement in the SCCM/ESICM Surviving Sepsis Campaign, Surviving Sepsis Campaign on the management of critically ill adults with Coronavirus Disease 2019, 2024 Focused Update: Guidelines on Use of Corticosteroids in Sepsis) and will not be involved in the AGREE II assessment of the related guidelines. Kimia Honarmand is involved in ongoing updates of the Surviving Sepsis Campaign.

## Supporting information


**APPENDIX S1:** Supporting information.

## Data Availability

All data will be included in the final manuscript and available in the original published guidelines.
