# Supplementary material for: A systematic methodological evaluation of sepsis guidelines: Protocol for quality assessment and consistency of recommendations
Source: Acta Anaesthesiol Scand. 2025 May 13;69(6):e70036. doi: 10.1111/aas.70036 (PMC12070244; doi:10.1111/aas.70036)
Supplement: Supplementary file 1 — APPENDIX S1: Supporting information. [file AAS-69-0-s001.docx]

**Supplementary Appendix**

**A** **Systematic Methodological Evaluation of Sepsis Guidelines: Protocol for Quality Assessment and Consistency of Recommendations**

Marwa Amer^1,2 †^, Morten Hylander Møller ^3,4^, Anders Granholm^3,5^, Haifa F. Alotaibi^6^, Shadan Al Muhaidib^7^, Zainab Al Duhailib^2,8^, Amr Arafat^6^, Michelle S Chew^9^, Marius Rehn^10,11,12^, Martin Ingi Sigurðsson^13,14^, Maija-Liisa Kalliomäki^15^, Klaus T. Olkkola^16^, Ville Jalkanen^17^, Wojciech Szczeklik^18,19^, Hassan M. Alshaqaq^20^, Kimberley Lewis ^21,22,23^, Kallirroi Laiya Carayannopoulos^21,22,23^**,** Kimia Honarmand^22,24^, Dipayan Chaudhuri^21,22^**,** Mustafa Alquraini^25^, Yasser S. Amer^26,27,28,29,30^, Fayez Alshamsi^31^, Waleed Alhazzani^6,32,33^

^1^Medical/Critical Pharmacy Division, King Faisal Specialist Hospital and Research Center, Riyadh, Saudi Arabia

^2^College of Medicine, Alfaisal University, Riyadh, Saudi Arabia

^3^Department of Intensive Care, Copenhagen University Hospital — Rigshospitalet, Copenhagen, Denmark

^4^Department of Clinical Medicine, University of Copenhagen, Copenhagen, Denmark

^5^Section of Biostatistics, Department of Public Health, University of Copenhagen, Copenhagen, Denmark

^6^Health Research center, Directorate General of Armed Forces Medical Services, Riyadh, Saudi Arabia

^7^Independent Researcher, Riyadh, Saudi Arabia

^8^Critical Care Medicine Department, King Faisal Specialist Hospital and Research Centre, Riyadh, Kingdom of Saudi Arabia

^9^Department of Perioperative Medicine and Intensive Care, Karolinska University Hospital, Stockholm, Sweden

^10^ Division of Prehospital Services, Air Ambulance Department, Oslo University Hospital, Oslo, Norway

^11^ The Norwegian Air Ambulance Foundation, Oslo, Norway

^12^ Institute of Clinical Medicine, University of Oslo, Oslo, Norway

^13^ Faculty of Medicine, University of Iceland, Iceland

^14^Division of Anaesthesia and Intensive Care Medicine, Landspitali—The National University Hospital of Iceland, Iceland

^15^Department of Anaesthesia, Tampere University Hospital, Tampere, Finland

^16^Department of Anaesthesiology, Intensive Care and Pain Medicine, University of Helsinki and Helsinki University Hospital, Helsinki, Finland

^17^ Department of intensive care medicine, Tampere University Hospital Tampere, Pirkanmaa, Finland

^18^Centre for Intensive Care and Perioperative Medicine, Jagiellonian University Medical College, Kraków, Poland

^19^Anesthesia and Intensive Care Department, 5th Military Hospital, Kraków, Poland

^20^Emergency Medicine Department, King Saud University Medical City, Riyadh, Saudi Arabia.

^21^Department of Medicine, McMaster University, Hamilton, ON, Canada

^22^Department of Health Research, Methods, Evidence, and Impact, McMaster University, Hamilton, ON, Canada.

^23^Research Institute of St Joseph’s Healthcare Hamilton, Hamilton, ON, Canada.

^24^Division of Critical Care, Department of Medicine, Mackenzie Health, Vaughan, ON, Canada.

^25^Emergency Medicine /Critical Care/ NeuroCritical Care Department, Almana Hospitals, Dammam, Saudi Arabia

^26^Pediatrics Department, King Khalid University Hospital, Riyadh, Saudi Arabia

^27^Clinical Practice Guidelines and Quality Research Unit, Quality Management Department, King Saud University Medical City, Riyadh, Saudi Arabia

^28^Research Chair for Evidence-Based Health Care and Knowledge Translation, King Saud University, Riyadh, Saudi Arabia

^29^Alexandria Center for Evidence-Based Clinical Practice Guidelines, Alexandria University, Alexandria, Egypt

^30^Adaptation Working Group, Guidelines International Network, Perth, Scotland

^31^Department of Internal Medicine, College of Medicine and Health Sciences, United Arab Emirates University, Alain, United Arab Emirates

^32^Department of Critical Care, College of Medicine, King Saud University, Riyadh, Saudi Arabia

^33^Critical Care and Internal Medicine Department, College of Medicine, Imam Abdulrahman Bin Faisal University, Saudi Arabia

**Running title:** Evaluating Sepsis Guidelines: A Protocol

†**Corresponding author:**

Marwa Amer, Critical Care Clinical Pharmacy Consultant, King Faisal Specialist Hospital & Research Center, Al Mathar Ash Shamali, Riyadh 11564, Saudi Arabia

Adjunct Assistant Professor- Alfaisal University- College of Medicine

Tel: +966114647272 Ext 70836 or 48030

Email address: [mamer@kfshrc.edu.sa](mailto:mamer@kfshrc.edu.sa), [amerdrmarwa@gmail.com](mailto:amerdrmarwa@gmail.com)

**Table of contents**

**Supplementary Appendix 1: Systematic Evaluation of Sepsis Guidelines – Literature Search**

**Supplementary Appendix 2: Instructions for Guideline Appraisal Using the AGREE II Instrument**

**Supplementary Figure 1: PRISMA 2020 Flow Diagram**

**Supplementary Table 1: Summary of AGREE II Domain Scores**

**Supplementary Table 2: Standardized Scores by Domains of AGREE II**

**Supplementary Table 3: AI-Assisted Review Prompts**

**Supplementary Table 4: Summary of AI Tools for Systematic Reviews**

**Supplementary Appendix 1**

**Systematic Evaluation of Sepsis Guidelines**

**– Literature Search**

**Date(s):** 2024 Dec 17 (as run)

**Limits:** 2004-current date

**Databases:** Ovid Medline [medall], Embase [oemezd]; The Cochrane Library (CENTRAL & CDSR); Specialized guideline platforms, as listed

**Filters:** Guidelines - Standard - MEDLINE, Embase [modified]; NOT Animal Only

**Search Output:** RIS (for Covidence)

**Syntax Definitions:** AU: Authors; CO: Contributor; KF: Keyword Heading Word; KW: Keyword Heading; PT: Publication Type; SH: Subject Heading; TI: Title; TW: Text Word, includes Title (TI) and Abstract (AB)

**Concept #1: Sepsis/Septic Shock**

exp *Systemic Inflammatory Response Syndrome/ [all]

exp *Cytokine Storm/ use oemezd

(bacter?emi* OR ((blood OR bloodstream OR blood-stream) ADJ (infect* OR poisoning*)) OR candid?emi* OR endotox?emi* OR endo-tox?emi* OR (endotoxic ADJ shock*) OR fung?emia OR parasite?emi* OR py?emia* OR pyoh?emia* OR septic OR sept#c?emia* OR sepsis OR (toxic ADJ shock*) OR tox?emi* OR urosepsis OR uroseptic* OR vir?emi*).ti,kf,kw. OR (bacter?emi* OR ((blood OR bloodstream OR blood-stream) ADJ (infect* OR poisoning*)) OR candid?emi* OR endotox?emi* OR endo-tox?emi* OR (endotoxic ADJ shock*) OR fung?emia OR parasite?emi* OR py?emia* OR pyoh?emia* OR septic OR sept#c?emia* OR sepsis OR (toxic ADJ shock*) OR tox?emi* OR urosepsis OR uroseptic* OR vir?emi*).ab./freq=2

(cytokine release syndrome? OR cytokine storm? OR hypercytokin?emi* OR hyper-cytokin?emi* OR ((hyperinflammat* OR hyper-inflammat*) ADJ2 (disease? OR disorder? OR syndrome? OR systemic*)) OR ((multisystem? OR multi-system? OR systemic*) ADJ inflammat* ADJ2 syndrome?)).ti,kf,kw. OR (cytokine release syndrome? OR cytokine storm? OR hypercytokin?emi* OR hyper-cytokin?emi* OR ((hyperinflammat* OR hyper-inflammat*) ADJ2 (disease? OR disorder? OR syndrome? OR systemic*)) OR ((multisystem? OR multi-system? OR systemic*) ADJ inflammat* ADJ2 syndrome?)).ab./freq=2

[Sepsis/Septic Shock]

**Filter: Guidelines - Standard - MEDLINE, Embase [modified]**

In: Search Filters Database. Ottawa: CADTH; 2024: <https://searchfilters.cda-amc.ca/link/25>. Accessed 2024-12-12.

(guideline OR practice guideline OR consensus development conference OR consensus development conference, NIH).pt. OR (guideline* OR standard? OR consensus* OR recommendat* OR statement?).ti. OR (expert opinion? OR practice parameter* OR position paper? OR position statement* OR policy statement* OR CPG OR CPGs OR best practice*).ti. OR (care ADJ2 (path OR paths OR pathway OR pathways OR map OR maps OR plan OR plans OR standard?)).ti. OR ((critical OR clinical OR practice) ADJ2 (path OR paths OR pathway OR pathways OR protocol*)).ti. OR (algorithm* ADJ5 (management OR pharmacotherap* OR therap* OR treatment* OR intervention*)).ti. OR (algorithm* ADJ5 (screening OR examination OR test OR tested OR testing OR assessment* OR diagnosis OR diagnoses OR diagnosed OR diagnosing)).ti. OR (guideline* OR standards OR consensus* OR recommendat*).au,co. [Guidelines]

**Filter: Animal Only (NOT’d)**

(exp animals/ OR exp animal experimentation/ OR exp animal experiment/ OR exp models animal/ OR nonhuman/ OR exp vertebrate/ OR exp vertebrates/) NOT (exp humans/ OR exp human experimentation/ OR exp human experiment/) [Animal Only]

Ovid

Database(s): **Embase**1974 to 2024 December 16**, Ovid MEDLINE(R) ALL**1946 to December 16, 2024**, EBM Reviews - Cochrane Central Register of Controlled Trials**November 2024**, EBM Reviews - Cochrane Database of Systematic Reviews**2005 to December 11, 2024
Search Strategy:

| **Line #** | **Searches** | **Results** |
| --- | --- | --- |
| 1 | exp *Systemic Inflammatory Response Syndrome/ [all] | 250153 |
| 2 | exp *Cytokine Storm/ use oemezd | 2772 |
| 3 | (bacter?emi* or ((blood or bloodstream or blood-stream) adj (infect* or poisoning*)) or candid?emi* or endotox?emi* or endo-tox?emi* or (endotoxic adj shock*) or fung?emia or parasite?emi* or py?emia* or pyoh?emia* or septic or sept#c?emia* or sepsis or (toxic adj shock*) or tox?emi* or urosepsis or uroseptic* or vir?emi*).ti,kf,kw. or (bacter?emi* or ((blood or bloodstream or blood-stream) adj (infect* or poisoning*)) or candid?emi* or endotox?emi* or endo-tox?emi* or (endotoxic adj shock*) or fung?emia or parasite?emi* or py?emia* or pyoh?emia* or septic or sept#c?emia* or sepsis or (toxic adj shock*) or tox?emi* or urosepsis or uroseptic* or vir?emi*).ab. /freq=2 | 423471 |
| 4 | (cytokine release syndrome? or cytokine storm? or hypercytokin?emi* or hyper-cytokin?emi* or ((hyperinflammat* or hyper-inflammat*) adj2 (disease? or disorder? or syndrome? or systemic*)) or ((multisystem? or multi-system? or systemic*) adj inflammat* adj2 syndrome?)).ti,kf,kw. or (cytokine release syndrome? or cytokine storm? or hypercytokin?emi* or hyper-cytokin?emi* or ((hyperinflammat* or hyper-inflammat*) adj2 (disease? or disorder? or syndrome? or systemic*)) or ((multisystem? or multi-system? or systemic*) adj inflammat* adj2 syndrome?)).ab. /freq=2 | 23682 |
| 5 | or/1-4 [Sepsis/Septic Shock] | 486320 |
| 6 | (guideline or practice guideline or consensus development conference or consensus development conference, NIH).pt. or (guideline* or standard? or consensus* or recommendat* or statement?).ti. or (expert opinion? or practice parameter* or position paper? or position statement* or policy statement* or CPG or CPGs or best practice*).ti. or (care adj2 (path or paths or pathway or pathways or map or maps or plan or plans or standard?)).ti. or ((critical or clinical or practice) adj2 (path or paths or pathway or pathways or protocol*)).ti. or (algorithm* adj5 (management or pharmacotherap* or therap* or treatment* or intervention*)).ti. or (algorithm* adj5 (screening or examination or test or tested or testing or assessment* or diagnosis or diagnoses or diagnosed or diagnosing)).ti. or (guideline* or standards or consensus* or recommendat*).au,co. [Guidelines] | 761694 |
| 7 | 5 and 6 | 4980 |
| 8 | (exp animals/ or exp animal experimentation/ or exp animal experiment/ or exp models animal/ or nonhuman/ or exp vertebrate/ or exp vertebrates/) not (exp humans/ or exp human experimentation/ or exp human experiment/) [Animal Only] | 12900079 |
| 9 | 7 not 8 | 4840 |
| 10 | limit 9 to yr="2004 -Current" [Sepsis/Septic Shock Guidelines, 2004-current, Animal Only studies removed]  Embase <1974 to 2024 December 16> 2317  Ovid MEDLINE(R) ALL <1946 to December 16, 2024> 1703  EBM Reviews - Cochrane Central Register of Controlled Trials <November 2024> 357  EBM Reviews - Cochrane Database of Systematic Reviews <2005 to December 11, 2024> 1 | 4378 |
| 11 | remove duplicates from 10 [Sepsis/Septic Shock Guidelines, 2004-current, Animal Only studies + duplicates removed]  Embase <1974 to 2024 December 16> 907 [846]  Ovid MEDLINE(R) ALL <1946 to December 16, 2024> 1698 [1697]  EBM Reviews - Cochrane Central Register of Controlled Trials <November 2024> 243 [219]  EBM Reviews - Cochrane Database of Systematic Reviews <2005 to December 11, 2024> 1 [0] | 2849 [2762, after final duplicates removed] |

*International Guideline Resources (searched separately)*

1. Best Practice Advocacy Centre New Zealand (bpacNZ) [New Zealand]: <http://www.bpac.org.nz/Default.aspx>

2024 Dec 18, 0 results

1. ECRI Guidelines Trust, ECRI Institute [United States]: <https://www.ecri.org/solutions/ecri-guidelines-trust>

2024 Dec 18, 6 results

1. Guidance and advice list, National Institute for Health and Care Excellence (NICE) [United Kingdom]: <https://www.nice.org.uk/guidance/published>

2024 Dec 18, 7 results

1. Guideline Central [United States], <https://www.guidelinecentral.com/guidelines/>

2024 Dec 18, 5 results

1. ICSI Guidelines, Institute for Clinical Systems Improvement [United States]: <https://www.icsi.org/guideline>

2024 Dec 18, 0 results

1. International guidelines library, Guidelines International Network (GIN) [United Kingdom]: <https://g-i-n.net/international-guidelines-library/>

2024 Dec 18, 11 results

1. SIGN guidelines, Scottish Intercollegiate Guidelines Network (SIGN) [United Kingdom]: <https://www.sign.ac.uk/our-guidelines>

2024 Dec 18, 1 result

**Literature Search and Study Selection**

Literature Search Methodology

The literature search was performed by an information specialist. Published literature was identified by searching the following bibliographic databases: Medline (1946‒ ), Embase (1974‒ ), Cochrane Central Register of Controlled Trials (Nov 2024), and Cochrane Database of Systematic Reviews (2005‒ ), via Ovid. All searches were run simultaneously as a multifile search and duplicates were removed in Ovid, then using manual deduplication in Endnote. The search strategy was comprised of both controlled vocabulary, such as the NLM’s MeSH (Medical Subject Headings), and keywords. International guideline resources were searched separately. The main search concept was sepsis/septic shock.

For the Ovid search, CADTH’s standard guideline filter was used, slightly modified (Guidelines - Standard - MEDLINE, Embase [modified]. In: Search Filters Database. Ottawa: CADTH; 2024: [https://searchfilters.cda-amc.ca/link/25. Accessed 2024-12-12](https://searchfilters.cda-amc.ca/link/25.%20Accessed%202024-12-12)), and animal only studies were removed, where possible. Retrieval was limited to a publication date of 2004 to current date. See Appendix 1 for the detailed search strategy. The initial search was completed on December 17-18, 2024.

**Appendix 1:** **Literature Search Strategy**

**Databases**

Ovid – Medline (1946 to December 16, 2024)

Ovid – Embase (1974 to 2024 December 16)

Ovid – Cochrane Central Register of Controlled Trials (November 2024)

Ovid – Cochrane Database of Systematic Reviews (2005 to December 11, 2024)

Note: Subject headings and search fields have been customized for each database. Additional duplicates were removed using bibliographic management software.

**Date of searches**: 2024 December 17-18

**Alerts**: None

**Search filters applied**: Guidelines - Standard - MEDLINE, Embase [modified]. In: Search Filters Database. Ottawa: CADTH; 2024: [https://searchfilters.cda-amc.ca/link/25. Accessed 2024-12-12](https://searchfilters.cda-amc.ca/link/25.%20Accessed%202024-12-12)

**Limits**: 2004 to 2024

**Database Search Strategy**

*Ovid*

| **Line #** | **Searches** | **Results** |
| --- | --- | --- |
| 1 | exp *Systemic Inflammatory Response Syndrome/ [all] | 250153 |
| 2 | exp *Cytokine Storm/ use oemezd | 2772 |
| 3 | (bacter?emi* or ((blood or bloodstream or blood-stream) adj (infect* or poisoning*)) or candid?emi* or endotox?emi* or endo-tox?emi* or (endotoxic adj shock*) or fung?emia or parasite?emi* or py?emia* or pyoh?emia* or septic or sept#c?emia* or sepsis or (toxic adj shock*) or tox?emi* or urosepsis or uroseptic* or vir?emi*).ti,kf,kw. or (bacter?emi* or ((blood or bloodstream or blood-stream) adj (infect* or poisoning*)) or candid?emi* or endotox?emi* or endo-tox?emi* or (endotoxic adj shock*) or fung?emia or parasite?emi* or py?emia* or pyoh?emia* or septic or sept#c?emia* or sepsis or (toxic adj shock*) or tox?emi* or urosepsis or uroseptic* or vir?emi*).ab. /freq=2 | 423471 |
| 4 | (cytokine release syndrome? or cytokine storm? or hypercytokin?emi* or hyper-cytokin?emi* or ((hyperinflammat* or hyper-inflammat*) adj2 (disease? or disorder? or syndrome? or systemic*)) or ((multisystem? or multi-system? or systemic*) adj inflammat* adj2 syndrome?)).ti,kf,kw. or (cytokine release syndrome? or cytokine storm? or hypercytokin?emi* or hyper-cytokin?emi* or ((hyperinflammat* or hyper-inflammat*) adj2 (disease? or disorder? or syndrome? or systemic*)) or ((multisystem? or multi-system? or systemic*) adj inflammat* adj2 syndrome?)).ab. /freq=2 | 23682 |
| 5 | or/1-4 [Sepsis/Septic Shock] | 486320 |
| 6 | (guideline or practice guideline or consensus development conference or consensus development conference, NIH).pt. or (guideline* or standard? or consensus* or recommendat* or statement?).ti. or (expert opinion? or practice parameter* or position paper? or position statement* or policy statement* or CPG or CPGs or best practice*).ti. or (care adj2 (path or paths or pathway or pathways or map or maps or plan or plans or standard?)).ti. or ((critical or clinical or practice) adj2 (path or paths or pathway or pathways or protocol*)).ti. or (algorithm* adj5 (management or pharmacotherap* or therap* or treatment* or intervention*)).ti. or (algorithm* adj5 (screening or examination or test or tested or testing or assessment* or diagnosis or diagnoses or diagnosed or diagnosing)).ti. or (guideline* or standards or consensus* or recommendat*).au,co. [Guidelines] | 761694 |
| 7 | 5 and 6 | 4980 |
| 8 | (exp animals/ or exp animal experimentation/ or exp animal experiment/ or exp models animal/ or nonhuman/ or exp vertebrate/ or exp vertebrates/) not (exp humans/ or exp human experimentation/ or exp human experiment/) [Animal Only] | 12900079 |
| 9 | 7 not 8 | 4840 |
| 10 | limit 9 to yr="2004 -Current" [Sepsis/Septic Shock Guidelines, 2004-current, Animal Only studies removed]  Embase <1974 to 2024 December 16> 2317  Ovid MEDLINE(R) ALL <1946 to December 16, 2024> 1703  EBM Reviews - Cochrane Central Register of Controlled Trials <November 2024> 357  EBM Reviews - Cochrane Database of Systematic Reviews <2005 to December 11, 2024> 1 | 4378 |
| 11 | remove duplicates from 10 [Sepsis/Septic Shock Guidelines, 2004-current, Animal Only studies + duplicates removed]  Embase <1974 to 2024 December 16> 907 [846]  Ovid MEDLINE(R) ALL <1946 to December 16, 2024> 1698 [1697]  EBM Reviews - Cochrane Central Register of Controlled Trials <November 2024> 243 [219]  EBM Reviews - Cochrane Database of Systematic Reviews <2005 to December 11, 2024> 1 [0] | 2849 [2762, after final duplicates removed] |

**International Guideline Resources**

*Best Practice Advocacy Centre New Zealand (bpacNZ) [New Zealand]*

<http://www.bpac.org.nz/Default.aspx>

Search terms – sepsis OR septic shock = 0 results

*ECRI Guidelines Trust, ECRI Institute [United States]*

<https://www.ecri.org/solutions/ecri-guidelines-trust>

Search terms – sepsis OR septic shock = 6 results

*Guidance and advice list, National Institute for Health and Care Excellence (NICE) [United Kingdom]*

<https://www.nice.org.uk/guidance/published>

Search terms – sepsis OR septic shock = 7 results

*Guideline Central [United States]*

<https://www.guidelinecentral.com/guidelines/>

Search terms – sepsis OR septic shock = 5 results

*ICSI Guidelines, Institute for Clinical Systems Improvement [United States]*

<https://www.icsi.org/guideline>

Search terms – sepsis OR septic shock = 0 results

*International guidelines library, Guidelines International Network (GIN) [United Kingdom]*

<https://g-i-n.net/international-guidelines-library/>

Search terms – sepsis OR septic shock = 11 results

*SIGN guidelines, Scottish Intercollegiate Guidelines Network (SIGN) [United Kingdom]*

<https://www.sign.ac.uk/our-guidelines>

Search terms – sepsis OR septic shock = 1 result

**We searched the following websites manually**

| Site | Description | Link |
| --- | --- | --- |
| NICE Guidelines (UK) | NICE publishes web-based guidelines. Some guidelines have PDFs, others require navigation to access recommendations. | <https://www.nice.org.uk/guidance/published> |
| Centers for Disease Control and Prevention (CDC) | CDC provides guidelines on sepsis management, often embedded in online resources rather than standalone documents. | <https://www.cdc.gov/sepsis/> |
| Australian Commission on Safety and Quality in Health Care (ACSQHC) | Australian guidelines, including sepsis-related ones, are available online but may not be downloadable. | <https://www.safetyandquality.gov.au/> |
| Canadian Critical Care Society (CCCS) | CCCS guidelines are available as online documents or web pages rather than downloadable files. | <https://www.canadiancriticalcare.org/> |
| Global Sepsis Alliance (GSA) | GSA hosts various sepsis-related resources and recommendations that are often web-based. | <https://www.global-sepsis-alliance.org/> |
| World Health Organization (WHO) | WHO provides global guidelines and recommendations for sepsis management. | <https://www.who.int/> |

**Supplementary Appendix 2**

**Instructions for Guideline Appraisal Using the AGREE II Instrument**

<https://www.agreetrust.org/help/>

<https://www.agreetrust.org/resource-centre/>

<https://www.agreetrust.org/my-agree/>


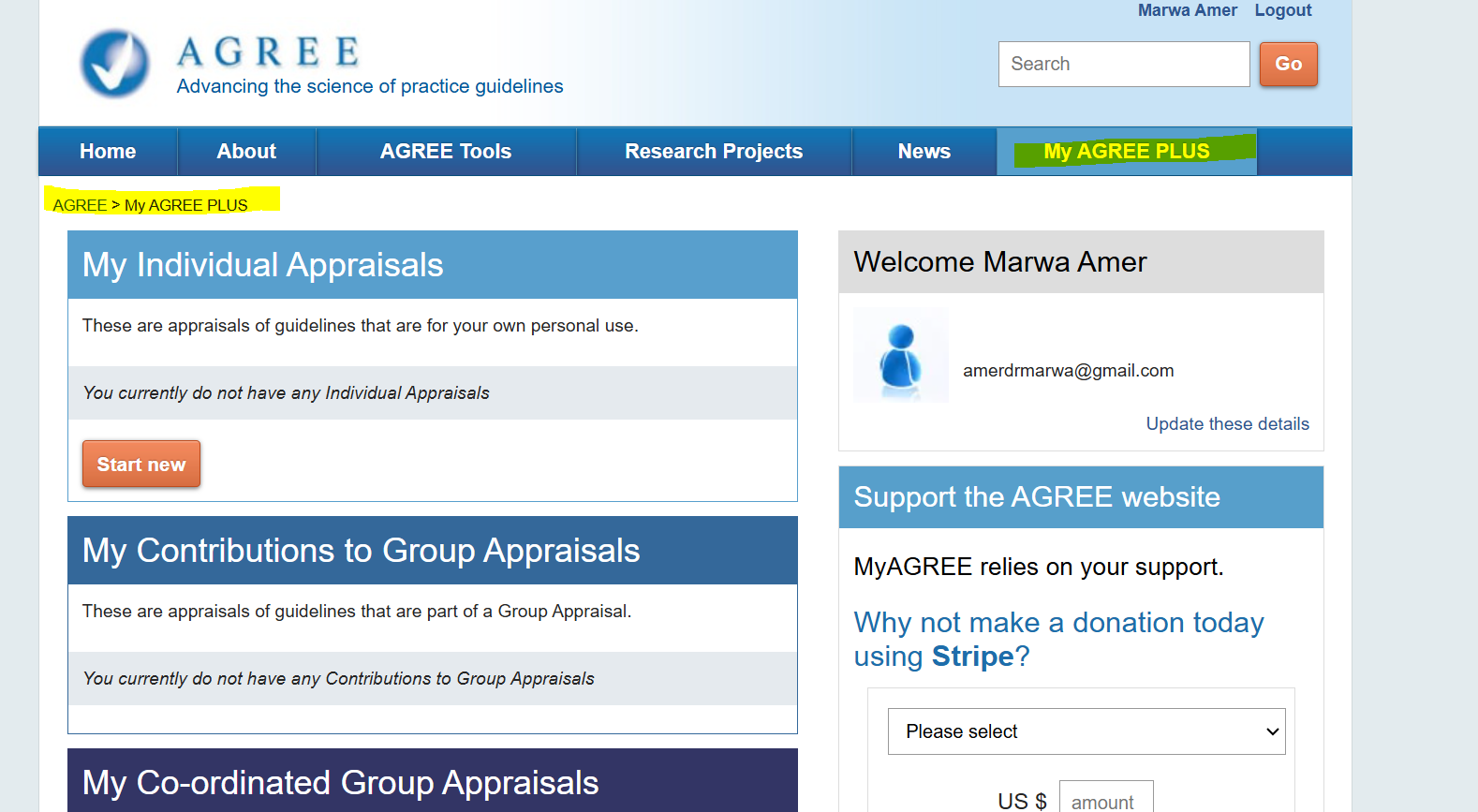


The AGREE PLUS platform, provided by the AGREE Trust, is a web-based tool designed to facilitate guideline appraisal using the AGREE II instrument. It provides a structured environment for conducting guideline quality assessments collaboratively

**1. Streamlining the Appraisal Process**

- Collaborative Scoring: AGREE PLUS allows multiple reviewers to independently score guidelines online. Each domain of the AGREE II instrument can be assessed systematically, ensuring consistency in methodology.
- Centralized Data Storage: All appraisals and comments are stored centrally on the platform, which reduces the likelihood of errors or lost data during the assessment phase.

**2. Improving Transparency**

- Traceability: Each reviewer’s scores and justifications for their ratings are clearly documented. This transparency makes it easier to identify areas of agreement or discrepancy.
- Audit Trail: The platform provides a clear record of how scores were determined, which can be included in systematic review to demonstrate rigor and adherence to methodology.

**3. Facilitating Consensus Building**

- Discrepancy Resolution: AGREE PLUS provides tools to compare scores among reviewers and highlight discrepancies. This feature streamlines discussions among the team to reach a consensus on contentious areas.
- Automatic Averages and Summary Scores: The platform can calculate domain and overall scores automatically, saving time and ensuring accuracy.

**4. Enhancing Training and Usability**

- User Guidance: AGREE PLUS offers integrated help features, including guidance on how to use the AGREE II tool and definitions for each domain.
- Practice Appraisals: It allows teams to conduct practice sessions to familiarize themselves with the tool and scoring methodology before formal appraisals.
- Conduct Pilot Appraisals: Use a few guidelines to conduct pilot assessments, allowing reviewers to practice and resolve any issues.

**5. Facilitating Reporting**

- Comprehensive Reports: Once appraisals are complete, AGREE PLUS generates detailed reports summarizing scores across all domains.
- Customizable Outputs: can tailor reports to include specific details such as domain scores, inter-rater reliability metrics, and reviewer comments.

**6. Time and Resource Efficiency**

- Ease of Use: The online platform reduces logistical challenges associated with manual scoring and aggregation.
- Global Accessibility: Reviewers from different locations can access the platform, enabling international collaboration without the need for in-person meetings.

**Training of AGREE II Tools**

Before conducting the guideline assessment, appraisers should complete a structured training to ensure consistent application of the AGREE II Instrument:

1. **AGREE II User’s Manual**: Each appraiser should review the AGREE II user manual to understand scoring nuances for each item and domain.
2. **AGREE PLUS Platform Registration**:
   - Register on **My AGREE PLUS** at [AGREE Trust](http://www.agreetrust.org/resource-centre/agree-plus/) to access training modules.
   - Complete the **AGREE II Online Training Tool** to practice scoring with sample guidelines.
3. **Practice Assessments**:
   - Conduct trial assessments on two clinical practice guidelines to develop scoring consistency. Discuss the results with experienced appraisers.

**Supplementary Figure 1. Proposed presentation of Preferred reporting items for systematic reviews and meta-analysis 2020 flow diagram showing the selection process of CPGs.**

**Identification of studies via other methods**

**Identification of studies via databases and registers**

Records identified from:

Websites (n = )

Organisations (n = )

Citation searching (n = )

etc.

Reports excluded:

Reason 1 (n = )

Reason 2 (n = )

Reason 3 (n = )

etc.

Reports assessed for eligibility

(n = )

Reports not retrieved

(n = )

Reports sought for retrieval

(n = )

Records excluded**

(n = )

Records screened

(n = )

Records identified from*:

Databases (n = )

Registers (n = )

Records removed *before screening*:

Duplicate records removed (n = )

Records marked as ineligible by automation tools (n = )

Records removed for other reasons (n = )

**Identification**

Reports not retrieved

(n = )

Reports sought for retrieval

(n = )

**Screening**

Reports excluded:

Reason 1 (n = )

Reason 2 (n = )

Reason 3 (n = )

etc.

Reports assessed for eligibility

(n = )

Studies included in review

(n = )

Reports of included studies

(n = )

**Included**

**Supplementary Table 1. Proposed presentation of Summary of AGREE II domain scores for clinical practice guidelines (n =   ).**

| **AGREE II Domains** | **Mean** | **SD** | **Median** | **IQR** | **Range** | **Domain Score ≥ 60%**  **n (%)** |
| --- | --- | --- | --- | --- | --- | --- |
| **Scope and purpose** |  |  |  |  |  |  |
| **Stakeholder involvement** |  |  |  |  |  |  |
| **Rigor of development** |  |  |  |  |  |  |
| **Clarity of presentation** |  |  |  |  |  |  |
| **Applicability** |  |  |  |  |  |  |
| **Editorial independence** |  |  |  |  |  |  |

**Abbreviations:** SD, standard deviation; IQR, interquartile range

**Supplementary Table 2. Proposed presentation of Standardized scores by domains of AGREE II (n = ).**

| **Guideline** | **Scope and purpose** | **Stakeholder involvement** | **Rigor of development** | **Clarity of presentation** | **Applicability** | **Editorial independence** | **Overall recommendation** |
| --- | --- | --- | --- | --- | --- | --- | --- |
| **CPG 1** |  |  |  |  |  |  |  |
| **CPG 2** |  |  |  |  |  |  |  |
| **CPG 3** |  |  |  |  |  |  |  |
| **CPG 4** |  |  |  |  |  |  |  |
| **Mean score** |  |  |  |  |  |  |  |
| **Median score** |  |  |  |  |  |  |  |

**Supplementary Table 3.** **Prompts for ChatGPT**

| **1. General Prompt for Guideline Overview**  **"Extract and organize the following fields from the document into a structured table. Ensure clarity and completeness based on explicit statements in the text.   1. Title 2. Authors 3. Year 4. Country 5. Target Population 6. Development Methodology (e.g., GRADE, consensus)"   **2. Core Recommendations Prompt**  **"Identify and summarize core recommendations provided in the guideline, focusing on the following areas:   1. Fluid resuscitation (type, timing, volume). 2. Antimicrobial therapy (type, timing, duration). 3. Vasopressor use (type, timing, target BP). 4. Corticosteroids (indications, dosage, duration). 5. Source control (strategies and timing). 6. Blood glucose management (targets and strategies). Present recommendations in a tabular format, specifying strength (weak vs strong) and evidence level, if available."**   **3. AGREE II Domain Evaluation Prompt**  **"Evaluate the provided guideline based on AGREE II domains, including:   1. Scope and Purpose—Assess clarity of objectives, health questions addressed, and population focus. 2. Stakeholder Involvement—Assess inclusion of relevant stakeholders and transparency of conflicts of interest. 3. Rigor of Development—Evaluate evidence collection, synthesis methods, and transparency of updates. 4. Clarity of Presentation—Assess formatting, language, and accessibility of recommendations. 5. Applicability—Evaluate practical implementation aspects, including resource requirements. 6. Editorial Independence—Check funding source transparency and mitigation of bias. Provide specific examples and highlight strengths/weaknesses for each domain."** |
| --- |

**Supplementary Table 4. Summary of the AI tools for systematic reviews**

| **AI Tool** | **Purpose** | **Strengths** | **Limitations** |
| --- | --- | --- | --- |
| **ChatGPT** | Research question generation, search strategy development, data extraction, and synthesis. | - Generates PICO-based research questions and custom search strings.  - Accessible and widely used.  - High screening accuracy (96% specificity, 93% sensitivity).  - Assists in academic writing and synthesis. | - Requires strong human oversight to ensure outputs align with research goals.  - Potential for compounding errors without validation. |
| **Rayyan** | Screening (title/abstract) | - Semi-automated tool with active learning for prioritization. - Web-based, supports collaboration.  - Frequently updated. | - Requires manual input for keywords and training data.  - Limited free features; premium features require payment. |
| **Elicit.org** | Literature search, data extraction, synthesis. | - Supports process-based ML approaches for literature searches.  - Strong focus on synthesis.  - Chain-of-thought prompting and fine-tuned models. | - Subscription-based for full functionality.  - Less validated for risk of bias assessments. |
| **SciSpace** | Literature search, data extraction, quality assessment. | - Uses NLP and transformer models for PDF analysis.  - Integrates citation management.  - Allows PDF interaction and validation prompts. | - Variability in data extraction accuracy.  - Requires manual oversight for synthesis validation. |
| **Covidence** | Screening, data extraction, and quality assessment. | - Streamlined workflow from screening to synthesis.  - Supports collaborative review processes. | - Requires subscription for full functionality after a trial period.  - Less customizable AI capabilities. |
| **RobotReviewer** | Data extraction and quality assessment. | - Automates extraction from RCTs and risk-of-bias evaluation.  - High accuracy (71–78%). | - Focuses only on RCTs, limiting broader application.  - Lower precision than Cochrane reviews. |
